# Ethosomes and Transethosomes for Mangiferin Transdermal Delivery

**DOI:** 10.3390/antiox10050768

**Published:** 2021-05-12

**Authors:** Maddalena Sguizzato, Francesca Ferrara, Supandeep Singh Hallan, Anna Baldisserotto, Markus Drechsler, Manuela Malatesta, Manuela Costanzo, Rita Cortesi, Carmelo Puglia, Giuseppe Valacchi, Elisabetta Esposito

**Affiliations:** 1 Department of Chemical, Pharmaceutical and Agricultural Sciences, University of Ferrara, I-44121 Ferrara, Italy; sgzmdl@unife.it (M.S.); hllsnd@unife.it (S.S.H.); crt@unife.it (R.C.); 2 Department of Neurosciences and Rehabilitation, University of Ferrara, I-44121 Ferrara, Italy; frrfnc3@unife.it; 3 Department of Life Sciences and Biotechnology, University of Ferrara, I-44121 Ferrara, Italy; bldnna@unife.it; 4 Bavarian Polymer Institute (BPI) Keylab “Electron and Optical Microscopy” University of Bayreuth, D-95440 Bayreuth, Germany; markus.drechsler@uni-bayreuth.de; 5 Department of Neurosciences, Biomedicine and Movement Sciences, University of Verona, I-37134 Verona, Italy; manuela.malatesta@univr.it (M.M.); manuela.costanzo@univr.it (M.C.); 6 Department of Drug and Health Sciences, University of Catania, Viale Andrea Doria 6, I-95125 Catania, Italy; capuglia@unict.it; 7 Animal Science Department, Plants for Human Health Institute, NC Research Campus, NC State University, Kannapolis, NC 28081, USA; 8 Department of Food and Nutrition, Kyung Hee University, Seoul 02447, Korea

**Keywords:** ethosomes, transethosomes, mangiferin, franz cell, antioxidants

## Abstract

Mangiferin is a natural glucosyl xanthone with antioxidant and anti-inflammatory activity, making it suitable for protection against cutaneous diseases. In this study ethosomes and transethosomes were designed as topical delivery systems for mangiferin. A preformulation study was conducted using different surfactants in association with phosphatidylcholine. Vesicle dimensional distribution was monitored by photon correlation spectroscopy, while antioxidant capacity and cytotoxicity were respectively assessed by free radical scavenging analysis and MTT on HaCaT keratinocytes. Selected nanosystems were further investigated by cryogenic transmission electron microscopy, while mangiferin entrapment capacity was evaluated by ultracentrifugation and HPLC. The diffusion kinetics of mangiferin from ethosomes and transethosomes evaluated by Franz cell was faster in the case of transethosomes. The suitability of mangiferin-containing nanovesicles in the treatment of skin disorders related to pollutants was investigated, evaluating, in vitro, the antioxidant and anti-inflammatory effect of ethosomes and transethosomes on human keratinocytes exposed to cigarette smoke as an oxidative and inflammatory challenger. The ability to induce an antioxidant response (HO-1) and anti-inflammatory status (IL-6 and NF-kB) was determined by RT-PCR and immunofluorescence. The data demonstrated the effectiveness of mangiferin loaded in nanosystems to protect cells from damage. Finally, to gain insight into the keratinocytes’ uptake of ethosome and transethosome, transmission electron microscopy analyses were conducted, showing that both nanosystems were able to pass intact within the cells.

## 1. Introduction

Mangiferin (MG) is a natural glucosyl xanthone found in both mango and papaya, possessing many pharmacological activities, such as hepatoprotective, anticarcinogenic, antidiabetic, and antiviral action against herpes simplex virus and poliovirus [1,2,3,4]. Notably, MG exerts potent antioxidant, antiapoptotic, and anti-inflammatory properties as demonstrated in various cell and animal models [5,6]. MG downregulates TNF-α expression under several conditions by suppressing NF-κB activity, a key pathway in an inflammatory reaction, which can be significantly induced by TNF-α [7]. Its ability to antagonize inflammatory reactions makes MG suitable for protection against cutaneous diseases such as contact dermatitis and psoriasis [8]. Moreover, MG could act as an anti-aging agent due to its ability to not only counteract reactive oxygen species (ROS) production induced by different challenges including UV exposure, but also to inhibit cutaneous collagenase and elastase activity [9]. Nonetheless, due to its scarce solubility in water and low bioavailability, MG requires specialized delivery systems that can entrap the molecule in a physiological environment and protect it against oxidative degradation.

An interesting technological strategy for MG application and delivery through the skin can be its loading in lipid-based nanovesicular systems. Particularly, ethosomes (E) are biocompatible nanovesicular systems based on phosphatidylcholine (PC), ethanol, and water, suitable for drug delivery across the skin. Indeed, ethosomal dispersions can solubilize lipophilic molecules in a double-layer phospholipid matrix, with high affinity with the biological membranes [10]. The peculiarity of E with respect to the classic liposomes is related to the presence of ethanol (20–45%) that improves the vesicle stability and the entrapment capacity of lipophilic drugs, enhancing the vesicle penetration potential [11]. Recent studies have demonstrated a transdermal potential ascribable to the penetration-enhancing synergistic properties of both PC and ethanol. Indeed, the E vesicle can cross the stratum corneum barrier due to the chemical affinity of PC with the stratum corneum lipids and to the ability of ethanol to perturb their organization, finally, allowing the transdermal passage of drugs through the skin [12,13,14]. Recent advances in vesicle nanotechnology led to the development of transethosomes (TE) that can be considered as a new generation of E, being vesicular systems, whose composition, mainly based on PC, ethanol, and water, is implemented by surfactants, employed as edge activators [15,16,17]. In TE the presence of surfactants added to PC might change the packing characteristics of the bilayer, leading to vesicles even more flexible than E [18]. Due to their remarkable transdermal potential, both E and TE can be proposed for the delivery of specific drugs for the treatment of several cutaneous pathologies [16,17].

The cutaneous tissue is continuously exposed to many exogenous stimuli, such as pollution, UV radiation, or oxidative compounds, including the ones derived from cigarette combustion [19,20,21]. The skin possesses non-enzymatic and enzymatic molecules, acting as potent antioxidants or oxidant-degrading systems. Nonetheless, under external stressors, skin defense mechanisms may not be powerful enough to counteract the deleterious effects of toxicants, leading to an increase of ROS in the skin, which can induce the development of dermatological diseases. For instance, the potent carcinogens contained in cigarettes cause various dermatological pathologies and disorders, such as squamous cell carcinoma, melanoma, psoriasis, atopic dermatitis eczema, acne, and skin aging, besides several chronic systemic diseases [22,23]. Indeed, cigarette smoke components can even pass through the epidermal barrier, readily penetrate skin cells, and reach the blood circulation, provoking systemic effects [24]. In the skin, these toxic compounds can (i) affect cellular redox homeostasis, (ii) influence keratinocyte proliferation and differentiation, and (iii) induce inflammatory responses. Given these assumptions, the administration of natural antioxidants through the skin represents an interesting strategy in the prevention or treatment of cutaneous ROS-mediated disorders. Recently, viscous glycerol-ethanol phospholipid nanosystems loaded with MG were proposed for the treatment of psoriasis [25]. The idea behind the present investigation is to evaluate the delivery of MG into the skin by E and TE for the eventual treatments of cutaneous disorders. Particularly, the first part of the work relies on a formulative study to select the composition suitable for MG entrapment. In the second part, the anti-inflammatory and antioxidant potential of selected nanosystems is studied in human keratinocytes exposed to cigarette smoke, while the uptake of E and TE in keratinocytes is evidenced by transmission electron microscopy (TEM).

## 2. Materials and Methods

### 2.1. Materials

Mangiferin, Mangifera indica, 1,3,6,7-Tetrahydroxyxanthone C2-β-D-glucoside (MG), polyoxyethylenesorbitan monolaurate, polysorbate 20 (TW_20_), polyoxyethylenesorbitan monooleate, polysorbate 80 (TW_80_), sorbitan monolaurate (SP_20_), sorbitane monooleate (SP_80_), dimethyldidodecylammonium bromide (DDAB), 2,2-Diphenyl-1-picrylhydrazyl (DPPH), nylon, mixed cellulose esters (MCE), and polytetrafluoroethylene (PTFE) membranes (diam. 25 mm, pore size 0.2 μm) were purchased from Merck, Sigma-Aldrich (Santa Louis, MO, USA). The soybean lecithin (90% phosphatidylcholine) (PC) was Epikuron 200 from Lucas Meyer, Hamburg, Germany. Solvents were HPLC grade and all other chemicals were analytical grade.

### 2.2. Mangiferin Solubility Evaluation

MG solubility was determined by saturating solvents (i.e., water, ethanol, methanol, ethanol/methanol 50:50, *v/v*, dimethyl sulfoxide, dimethyl sulfoxide/ethanol, 20:80, *v/v*, and propylene glycol/water 60:40, *v/v*) with an excess of the drug. The obtained saturated solutions were horizontally shaken at 150 rpm for 1 h in the dark at 25 °C. Afterward, 1 mL was withdrawn and filtered through a nylon filter membrane, 0.22 μm pore size, 25 mm diameter (Millipore-Sigma-Aldrich Merck, Darmstadt, Germany). MG concentration was determined by high-performance liquid chromatography (HPLC) analyses with the method below described.

### 2.3. Production of Ethosomes and Transethosomes

For the preparation of E and TE, PC (30% *w/w*) was solubilized in ethanol at 30 °C. Afterward, bidistilled water was slowly added to the ethanolic solution up to a final 70:30 (*v/v*) ratio, under continuous magnetic stirring at 750 rpm by an IKA Eurostar digital (IKA Labortechnik Janke & Kunkel, Staufen, Germany) at 22–25 °C. Magnetic stirring was maintained for 30 min in the dark [26]. In the case of TE, the surfactants TW_80_, TW_20_, SP_20,_ or SP_80_ were alternatively added to the PC ethanol solution, up to final surfactant concentrations 0.1–0.6% *w/w*, before adding water. MG containing E or TE, were prepared by solubilizing the drug (3.3 mg/mL) in the PC ethanol solution (E-MG) or in the surfactant PC ethanol solution (TE-MG) before the addition of water, obtaining a final MG concentration of 1 mg/mL.

### 2.4. Photon Correlation Spectroscopy (PCS)

The size distribution of vesicles was evaluated using a Zetasizer Nano S90 (Malvern Instr., Malvern, UK) with a 5 mW helium-neon laser and a wavelength output of 633 nm. Measurements were performed at 25 °C at a 90° angle and a run time of at least 180 s. Samples were diluted with bidistilled water in a 1:10 *v/v* ratio. Data were analyzed by the “cumulant” method [27]. Measurements were conducted thrice during 3 months after E and TE production. Zeta potential values were acquired by measuring the electrophoretic mobility according to the Helmholtz–Smoluchowski equation [28].

### 2.5. Cell Culture and Cytotoxicity Study

HaCaT cells were cultured in high glucose Dulbecco’s Modified Eagle’s Medium (DMEM) supplemented with 10% FBS, 100 U/mL penicillin, and 100 μg/mL streptomycin. All cell cultures were performed at 37 °C in 5% CO_2_ and 95% air. Keratinocytes were grown in 96-well plates at a density of 2 × 10^4^ cells/well in 200 μL of media for MTT assay and in 6 cm^2^ Petri dishes at a density of 1.5 × 10^6^ cells in 3 mL of media for real-time PCR. Seeded cells were exposed to unloaded and MG-loaded formulations at various MG concentrations, ranging from 5 to 50 μM, for 24 h. After complete removal of the treatment to avoid any color interference, 50 μL of serum-free media and 50 μL MTT (0.5 mg/mL) were added and incubated for 3 h. The insoluble purple formazan crystals were then dissolved in 100 μL of DMSO at 37 °C for 15 min. After shaking, the solution absorbance was measured with a spectrophotometer at 590 nm, using 670 nm as a reference wavelength, and, thus, converted into a percentage of viability [29].

### 2.6. Mangiferin Content of Ethosomes and Transethosomes

The entrapment capacity (EC) of MG in E-MG and TE-MG has been determined by ultracentrifugation and HPLC analyses; specifically, 500 μL samples were loaded in a centrifugal filter (Microcon centrifugal filter unit YM-10 membrane, NMWCO 10 kDa, Sigma-Aldrich, St. Louis, MO, USA) and subjected to ultracentrifugation (Spectrafuge™ 24D Digital Microcentrifuge, Woodbridge, NJ, USA) at 4000 rpm for 15 min. One hundred microliters of E-MG or TE-MG in the supernatant were diluted with 900 μL of dimethyl sulfoxide and maintained under magnetic stirring for 30 min [30], while the filtered aqueous phase of E-MG and TE-MG was simply withdrawn from the lower part of the centrifugal filter unit. After filtration of the lipidic and aqueous phases by nylon syringe filters (0.22 μm pores), the amount of MG was quantified by HPLC, as above reported. The EC was determined as follows:

EC = MG/T_MG_ × 100 (1)

where MG corresponds to the amount of the drug measured by HPLC and T_MG_ is the total amount of MG employed for E-MG and TE-MG production.

### 2.7. Antioxidant Activity (2,2-Diphenyl-1-Picrylhydrazyl Assay)

MG-loaded E and TE were tested to evaluate the scavenging activity of DPPH radicals in accordance with a previously described modified procedure [31]. This in vitro radical-scavenging assay is widely used for a quick assessment of antioxidant capacity and is particularly ideal for phenolic compounds. The DPPH test can evaluate the ability of an antioxidant substance to donate hydrogen to convert the stable free radical DPPH into 1,1-diphenyl-2-picrylhydrazyl. This reaction is accompanied by a colorimetric variation (from deep purple to yellow if the tested substance reacts with the radical) which can be monitored by UV spectrophotometer (UV-31 SCAN ONDA, Sinergica, Milano, Italy) at 517 nm. The radical inhibition percentage is calculated using the following equation:

DPPH radical-scavenging capacity (%) = [1 − (A_1_ − A_2_)/A_0_] × 100% (2)

where A_0_ was the absorbance of the control (without sample), A_1_ was the absorbance in the presence of the sample, and A_2_ was the absorbance without DPPH.

Seven hundred and fifty microliters of each sample (E-MG, TE-MG, or MG solution) diluted in EtOH-DMSO (80:20, *v/v*) at different concentrations were added to the DPPH ethanolic solution (1.5 mL) and the absorbance was measured by UV-Vis spectrophotometer. The IC_50_ values were calculated from the results, determined by regression analysis of the results obtained at different sample concentrations, and expressed as µg/mL.

### 2.8. Cryo-Transmission Electron Microscopy (Cryo-TEM)

In order to vitrify samples, a 2 µL droplet was put on a lacey carbon-filmed copper grid (Science Services, Munich, Germany) for 30 s [32]. Subsequently, blotting paper was used to remove most of the liquid, resulting in a thin film stretched over the lace holes. The specimens were instantly shock frozen by rapidly immersing them into liquid ethane cooled to approximately 90 K by liquid nitrogen in a temperature-controlled freezing unit (Zeiss Cryobox, Carl Zeiss Microscopy GmbH, Jena, Germany). All the steps of sample preparation were conducted at a controlled temperature. After specimen freezing, the remaining ethane was removed by blotting paper. The vitrified specimen was then transferred to a Zeiss/Leo EM922 Omega EFTEM (Zeiss Microscopy GmbH, Jena, Germany) transmission electron microscope using a cryoholder (CT3500, Gatan, Munich, Germany). The temperature of samples was maintained below 100 K during the examination. Specimens were examined with reduced doses of about 1000–2000 e/nm^2^ at 200 kV. Images were recorded by a CCD digital camera (Ultrascan 1000, Gatan, Munich, Germany) and analyzed using a GMS 1.9 software (Gatan, Munich, Germany).

### 2.9. In Vitro Diffusion Experiments

Franz cells associated with nylon, MCE, or PTFE membranes (pore size 0.2 μm) were employed to evaluate the diffusion of MG. Namely, the membranes were hydrated in distilled water for 1 h before being placed in Franz-type diffusion cells produced by Vetrotecnica (Padova, Italy) [33]. The membrane surface area exposed to diffusion was 0.78 cm^2^ (1 cm diameter orifice). Franz cells consisted of a lower receptor compartment and an upper donor compartment sealed to avoid evaporation during the experiments. Five milliliters of bidistilled water or ethanol/water (30:70 or 50:50, *v/v*) mixture was poured into the lower section, stirred at 500 rpm by a magnetic bar, and maintained at 32 ± 1 °C during all the experiments [34]. Roughly 1 g of MG containing dosage form was placed in the donor compartment on the membrane surface. Namely, MG solution (0.7 mg/mL) in ethanol/water (30:70 *v/v*) (Sol-MG), E-MG (1 mg/mL) or TE-MG (1 mg/mL) were employed. Two hundred microliters of receptor phase were withdrawn at predetermined time intervals (0.5–6 h) and analyzed for MG content by HPLC as reported below. Each removed sample was replaced with an equal volume of simple receptor phase. The concentrations of MG were determined six times in independent experiments. The mean values ± standard deviations were calculated and plotted as a function of time. From the linear portion of the accumulation curve, the fluxes were obtained, referring to the slopes of the regression line (angular coefficient). Diffusion coefficients were calculated according to Equation (3).

D = F/[MG] (3)

where D is the diffusion coefficient, F is the flux, and [MG] is the MG concentration in the dosage form, expressed in mg/mL.

### 2.10. HPLC Analysis

HPLC analyses were performed by a two-plunger alternative pump (Agilent Technologies 1200 series, Santa Clara, CA, USA), a UV-detector operating at 254 nm, and a 7125 Rheodyne injection valve with a 50 μL loop. A stainless-steel C-18 reverse-phase column (15 × 0.46 cm) packed with 5 μm particles (Platinum C18, Apex Scientific, Alltech, KY, USA) was eluted with a mobile phase containing methanol/water 60:40 *v/v*, pH 4.0 at a flow rate of 1 mL/min.

### 2.11. Cigarette Smoke (CS) Exposure

HaCaT cells were pre-treated with unloaded or MG-loaded formulations, using the MG concentration selected by cytotoxicity studies. After 24 h, cells were exposed to cigarette smoke (CS) for 40 min, using 1 research cigarette (12 mg tar, 1.1 mg nicotine). CS was generated by a vacuum pump that could burn the research cigarette, as previously described [35]. Untreated cells were exposed to filtered air to compare the damage of CS, while untreated cells exposed to CS were used as control. After exposure, the culture medium was changed, and the cells were incubated at 37 °C in a humidified 5% CO_2_ atmosphere. Then, RNA was collected at different time points (i.e., 2 h and 6 h post-exposure) as reported below [36].

### 2.12. RNA Extraction and Quantitative Real-Time PCR

For RNA extraction of HaCaT cells, total RNA was extracted following the phenol-chloroform extraction protocol described by Toni et al. [37], adopting some modifications. Briefly, HaCaT cells were washed twice with PBS and then suspended in 500 μL of PureZOL™ RNA Isolation Reagent (Biorad, Hercules, CA, USA). After adding 100 μL of chloroform, the different organic phases were separated by centrifuging the cell suspension at 12,000 rpm for 15 min at 4 °C. The upper aqueous phase containing RNA was collected in new Eppendorf tubes. After repeating the chloroform step twice, 250 μL of methanol was added to the aqueous phase containing RNA to precipitate the RNA pellet by centrifuging at 12,000 rpm for 10 min at 4 °C. The RNA pellet was washed 3 times in 1 mL of 75% ethanol centrifuging at 12,000 rpm for 10 min at 4 °C. The Eppendorf tubes were left open at RT for 3–5 min to evaporate ethanol and then the RNA pellet was suspended in 25 μL of nuclease-RNAse-free water. RNA concentration was measured using the Shimadzu BioSpec-nano spectrophotometer (Shimadzu Biotech, Duisburg, Germany). Next, cDNA was generated from 1 μg of total RNA, using the iScript cDNA Synthesis Kit (Biorad, Hercules, CA, USA). To evaluate the mRNA levels of HO-1 and IL-6 genes, quantitative real-time PCR was performed using SYBR^®^ Green Master Mix (Biorad, Hercules, CA, USA) on a CFX Connect Real-Time PCR System (Biorad, Hercules, CA, USA) following the manufacturer’s protocol. Gene expression was quantified by obtaining the number of cycles to reach a predetermined threshold value in the intensity of the PCR signal (CT value). As the reference, gene RPL11 was used, while samples were compared using the relative cycle threshold (CT). After normalization, quantitative relative gene expression was calculated by the 2−ΔΔCt method [38]. Primer sequences used for analysis of gene expression are as follows: HO-1 fwd, TTGCTTTGGCGAGCTCTTTT; HO-1 rev, TCTGATGCCAAAACACCCCA; IL-6 fwd, TAGGACTGGAGATGTCTGAGGCT; IL-6 rev, GACCGAAGGCGCTTGTGGA; RPL11 fwd, ACTTCGCATCCACAAACTCT; RPL11 rev, TGTGAGCTGCTCCAACACCTT.

### 2.13. Immunocytochemistry

HaCaT cells were grown on coverslips at a density of 1 × 10^5^ cells/mL. After 24 h of pre-treatment with unloaded or MG-loaded formulations, cells were exposed to CS as above described, and analyzed 1 h post-exposure. Afterward, cells were fixed in 4% paraformaldehyde for 10 min at 22–25 °C. After cell permeabilization at 22–25 °C for 5 min with PBS containing 0.2% Triton X-100, the coverslips were blocked in PBS containing 1% BSA for 1 h. Then, the cells were incubated overnight with primary antibody for NF-kB (8242, Cell Signaling, Danvers, MA, USA) 1:400, in PBS containing 0.5% BSA at 4 °C. Coverslips were washed and incubated with appropriate secondary antibody (1:100) for 1 h at RT. Nuclei were stained with 1 μg/mL DAPI (Sigma-Aldrich, Merck, Darmstadt, Germany) for 1 min. Coverslips were mounted onto glass slides using anti-fade mounting medium 1,4 diazabicyclooctane (DABCO) in glycerin and examined by a Leica light microscope equipped with epifluorescence at 40× magnification. Negative controls for the immunostaining experiments were processed omitting the primary antibody. Images were acquired and analyzed with Leica software [30,39].

### 2.14. Transmission Electron Microscopy

Cells treated with E or TE for 24 h were fixed with 2.5% (*v/v*) glutaraldehyde and 2% (*v/v*) paraformaldehyde in 0.1 M phosphate-buffered, pH 7.4, for 2 h at 4 °C. Cells were then post-fixed with 1.5% potassium ferrocyanide and 1% osmium tetroxide for 1 h, dehydrated with acetone, and embedded in Epon resin [40]. Ultrathin sections were observed using a Philips Morgagni transmission electron microscope (FEI Company Italia S.r.l., Milan, Italy) operating at 80 kV and equipped with a Megaview II camera for digital image acquisition. All images were processed using Paint Shop Pro software (JASC Software Inc., Eden Praire, MN, USA).

### 2.15. Statistical Analysis

All statistical analyses have been calculated by repeated-measures analysis of variance (ANOVA) and the Dunnett comparison procedure. The software Prism 6.0, Graph Pad Software Inc. (La Jolla, CA, USA) has been employed. Probability values (*p*) less than 0.05 were regarded significant in this study.

## 3. Results and Discussion

### 3.1. Preparation and Characterization of Ethosomes and Transethosomes

To design a nanotechnological formulation suitable for MG delivery through the skin, a preliminary screening was performed, evaluating the drug solubility in different solvents and solvent mixtures. The results reported in Table 1, show very slight solubility values, the highest of which was in the case of dimethyl sulfoxide.

Despite its solubilizing power, dimethyl sulfoxide causes burning, itching, and strong allergic reactions on contact with the skin, while ethanol is more acceptable and non-toxic when topically applied [41,42]. Therefore, E and TE were chosen to investigate MG delivery, because they are nanosystems based mainly on ethanol, water, and PC, which is a glycerophospholipid that possesses penetration enhancer properties due to its high affinity with stratum corneum components [43]. Thanks to the solubilizing power of PC, MG solubility reached 3.3 mg/mL in PC ethanolic solution (3% *w/v*). On this basis, a formulative study was conducted, using PC in the case of E, or different surfactants in association with PC in the case of TE. Particularly, E was prepared by slow addition of water to a PC ethanol solution under stirring, resulting in milky and homogeneous E dispersions whose final PC concentration was 0.9% *w/v*. This concentration was selected based on a previous study demonstrating its suitability to adequately entrap lipophilic drugs, while maintaining the vesicle size stability [30]. TE dispersions were similarly obtained solubilizing surfactants in the PC solution before the addition of water. Particularly, hydrophilic (TW_20_ and TW_80_) and lipophilic (SP_20_ and SP_80_) non-ionic surfactants were employed, as well as the cationic DDAB, as reported in Table 2.

The cationic surfactant DDAB was employed to confer a positive charge to vesicles, to possibly enhance their cutaneous permeation properties, by electric attraction with the negatively charged skin surface. In all cases, TE homogeneous dispersions were obtained, which were milky in the case of TE2 and TE3, translucent in the case of TE1–TE6, and almost transparent in the case TE7–TE9 (Supplementary Figure S1). Indeed, the presence of TW_80_ or DDAB resulted in dispersions whose transparency was directly proportional to the surfactant concentration. The influence of composition on vesicle size distribution was studied by PCS (Table 3). Surfactants are thought to intercalate with the vesicle lipid bilayer, rearranging it, thus, affecting the vesicle mean diameter, as a function of the type and concentration of employed surfactant [18]. Z-Average mean diameters spanned between 82 and 411 nm, while dispersity indexes were lower than 0.2, indicating a homogeneous size distribution, mostly characterized by the presence of one peak. The largest mean diameters were found in the case of TE1 and TE2, produced in the presence of TW_20_ and SP_20_, while the smallest were found in TE7–TE9, produced in the presence of DDAB. In the case of TE4–TE6 and TE7–TE9, as expected, the higher the surfactant concentration, the lower the mean diameter.

In order to predict the stability of E and TE, the zeta potential was evaluated because this parameter reflects the degree of repulsion between the vesicles in the dispersion. In the case of E, zeta potential values, reported in Table 3, were negative, due to the presence of ethanol, which provides a negative charge on the vesicle surface [44]. Conversely, in the case of TE7–TE9, the cationic surfactant presence conferred a positive charge to the vesicle surface, leading to the highest positive zeta potential values in terms of absolute values. Notably, TE7–TE9 showed the lowest mean diameters since the electric repulsion of these highly charged vesicles prevented aggregation phenomena. On the other hand, TE1–TE4 and TE–6 produced in the presence of T_20_, SP_20,_ SP_80_, and T_80_ 0.6% displayed negative zeta potential values, with the lowest absolute values. In these latest cases, it is likely that the attraction of the vesicles could overcome repulsion, possibly leading to aggregation phenomena over time.

**Figure 1:**
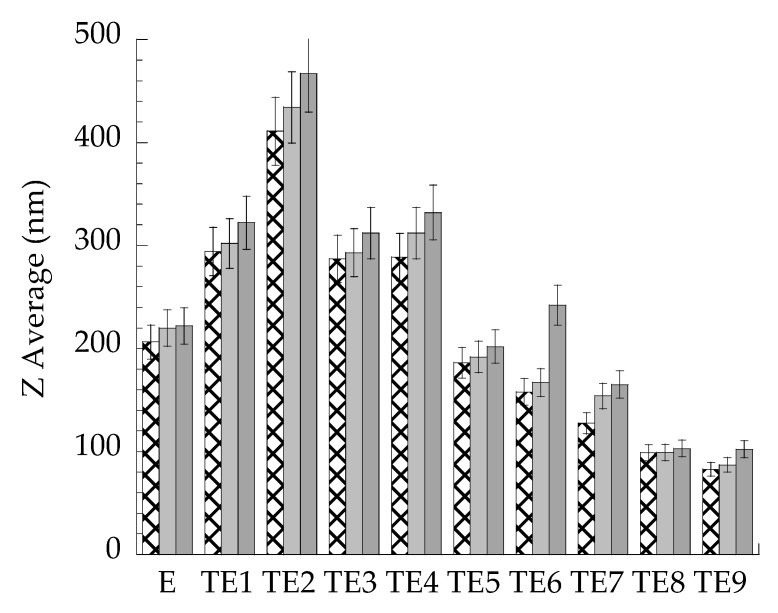
Effect of storage on vesicle mean diameter. E and TE were stored at 25 °C for 1 (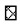), 2 (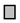) and 3 (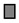) months. Diameters were measured by PCS and expressed as Z average.

To verify these hypotheses and to select the formulations suitable for MG loading, vesicle size stability was investigated, measuring Z Average mean diameters by PCS during the 3 months after preparation. As shown in Figure 1, the vesicle mean diameter was almost stable in the case of E, TE5, and TE8, while an increase of mean diameters was particularly appreciable in the case of TE2 and TE6, possibly due to vesicle fusion and aggregation under storage. In this regard, some authors described a high tendency of vesicles stabilized by SP to aggregate, in reason of strong cohesive forces between the hydrophobic vesicles [45].

Based on the obtained stability data and considering that smaller vesicles have a higher chance to penetrate through the skin than larger ones, E, TE5, and TE8 were selected because they are characterized by higher dimensional stability and smaller mean diameters.

### 3.2. Preparation and Characterization of Ethosomes and Transethosomes Loaded with MG

E-MG, TE5-MG, and TE8-MG were easily prepared by solubilizing MG in the PC ethanol solution before water addition, resulting in homogeneous milky or translucent dispersions, like the corresponding unloaded ones (Supplementary Figure S1). As reported in Table 3, MG slightly affected Z average mean diameter and zeta potential, compared to vesicles produced in the absence of the drug. To evaluate the entrapment capacity of MG in E-MG, TE5-MG, and TE8-MG, the lipid phase was separated from the aqueous one by ultracentrifugation, and dissolved with dimethyl sulfoxide to promote vesicle disaggregation and MG solubilization. The quantification of MG in both phases confirmed the total recovery of the drug in the dispersion, suggesting that the production modalities avoided MG loss on mechanical devices and preserved the drug from possible thermal or light degradation. It is noteworthy that MG solubility in both E and TE reached 1 mg/mL, thereby 10-fold higher with respect to its solubility in water (Table 1). As expected, MG was mostly associated with the lipid phase, especially in the case of E-MG, as reported in Table 4. Nonetheless, MG was partly found in the aqueous phase (32%, 37%, and 43% for E-MG, TE5-MG, and TE8-MG, respectively). The different EC of MG should be ascribed to its possible localization in the bilayer at the polar–nonpolar interface. Since surfactant molecules are probably intercalated into the bilayer, they could alter the packing density of the bilayers and the permeability of the vesicle to the entrapped compounds, finally affecting the entrapment capacity [46].

In order to compare MG antioxidant capacity, the DPPH free radical scavenging activity of E-MG, TE5MG, TE8-MG, and MG solution was evaluated. The IC_50_ values, reported in Table 4, suggest that the antioxidant activity was better retained in the case of TE5-MG and E-MG, while TE8-MG displayed the lowest antioxidant activity.

### 3.3. Cytotoxicity Evaluation

**Figure 2:**
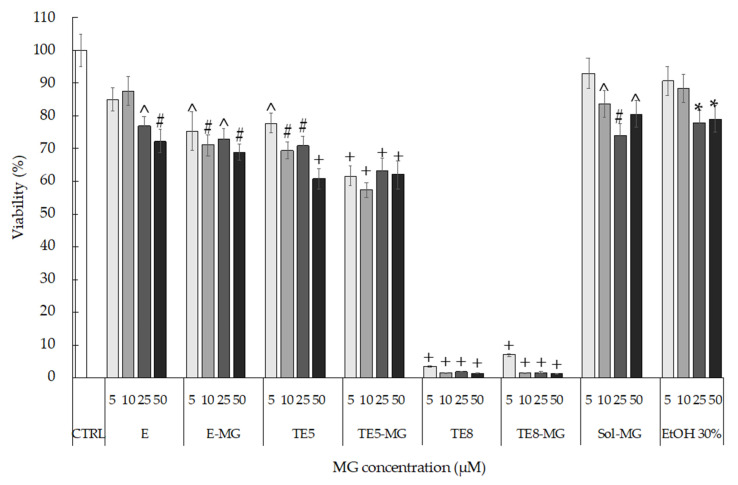
HaCaT cell viability evaluated by MTT test after 24 h of treatment with E, TE5, and TE8 produced in the absence and presence of MG. MG ethanol solution (Sol-MG) and sole ethanol (EtOH) were tested as a control. Data are given as mean ± SD, representative of three independent experiments with at least three technical replicates each time. * *p* < 0.05, ^ *p* < 0.01, # *p* < 0.001, + *p* < 0.0001 vs. CTRL sample by ANOVA.

To select a suitable carrier for MG based on biocompatibility, the in vitro cytotoxicity of E, TE5, and TE8, produced in the absence and presence of MG, was assessed by MTT in HaCaT cells, at the concentrations of E and TE ranging from 5 to 50 µM (referring to loaded MG). From the obtained results, depicted in Figure 2, E and TE5 showed more than 60% cell viability up to the highest concentration (50 µM). Moreover, it should be underlined that the presence of MG did not affect the toxicity of the vehicle, because no significant differences were detected between empty and loaded formulations. However, major toxicity has been found in the case of TE8. At all concentrations tested, both the vehicles and the loaded formulations caused almost complete cell death, revealing the toxic effect of DDAB on cells.

The cytotoxicity of DDAB could be ascribed to the cationic surfactant capability of solubilizing lipid membranes, changing their barrier capacity, and finally leading to cell lysis [47,48]. Based on IC_50_ values and cytotoxicity data, TE8 and TE8-MG were not considered for further experiments, while the 10 µM concentration of MG was selected for the evaluation of the antioxidant and anti-inflammatory potential of E-MG, TE5-MG, and Sol-MG.

### 3.4. Morphological Characterization

**Figure 3:**
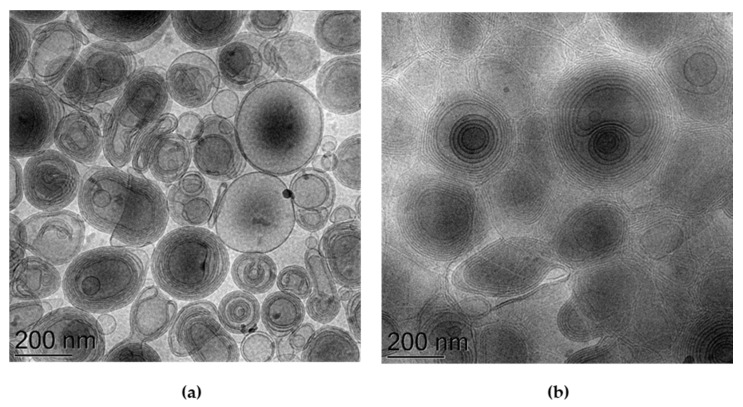
Cryo-transmission electron microscopy images (Cryo-TEM) of E-MG (**a**) and TE5-MG (**b**). Bar corresponds to 200 nm.

Figure 3 reports representative images of E-MG and TE5-MG samples, visualized by cryo-TEM, to gain insight into their morphologies. In both cases, spherical and ovoid multilamellar vesicles are detectable. Notably, in the case of the E-MG image, the contrast difference between the vesicles and the aqueous phase is higher (Figure 3a) compared to TE5-MG (Figure 3b) where the vesicles are close together. The multilamellar organization, typical of ethosome vesicles, is due to the packing organization of PC in the presence of water, forming hydrophobic double layers associated with the PC chains, delimiting hydrophilic domains, associated with the polar heads of PC, inside and outside the vesicles. In the case of TE5-MG, TW_80_ is thought to alter the vesicle bilayer, positioning it with the head group oriented towards the head group of PC and the oleic chain aligned parallel to the PC acyl chains [49].

### 3.5. MG Diffusion Kinetics

**Figure 4:**
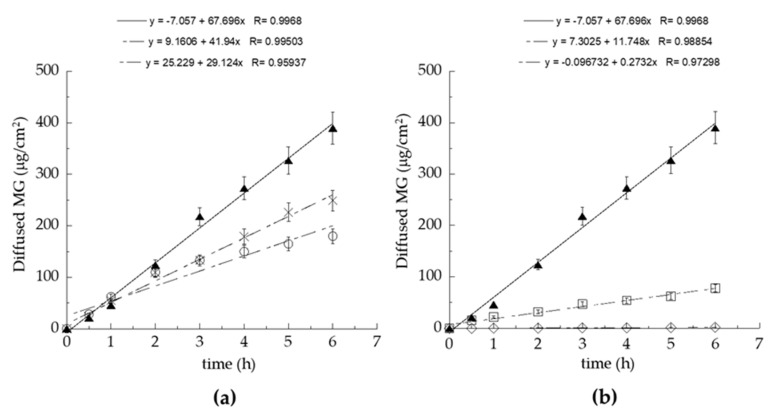
Effect of membranes (**a**) and receiving phases (**b**) on MG diffusion kinetics. Experiments were performed using Franz cells. (**a**) nylon (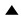), MCE (**×**), PTFE (o) membranes and ethanol/water 50:50, *v/v* as receiving phase; (**b**) ethanol/water 50:50, *v/v* (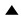), ethanol/water 30:70, *v/v* (**□**), water (**◊**) as receiving phases and nylon as membrane. Data are the mean of 6 independent experiments ± s.d.

In order to study MG diffusion kinetics from E-MG and TE5-MG, an in vitro system based on Franz cells associated with synthetic membranes was employed. Even though synthetic membranes are less predictable with respect to natural ones, their use is preferable for ethical reasons and indicated in formulative studies to gain information about the structural parameters affecting drug diffusion through the dosage form [29]. It should be considered that the supramolecular structure of the vehicle directly influences drug diffusion. In addition, since the diffusion results are strongly affected by experimental parameters, such as the employed membrane and the receiving phase, the choice of in vitro conditions is crucial to obtain reproducible and reliable diffusion information [50]. Therefore, a preliminary screening was performed to select the type of membrane and receiving phase, evaluating the diffusion of MG in ethanol solution (Sol-MG, 0.7 mg/mL). Particularly, nylon, MCE, and PTFE membranes (pore size 0.2 μm) were employed using ethanol/water 50:50, *v/v* as receiving phase (Figure 4a). The nylon membrane was selected because it could achieve the fastest MG diffusion, followed by MCE and PTFE; indeed, the membrane employed in the Franz cell test should not act as rate-limiting for drug diffusion [50]. As a second parameter, the type of receiving phase was evaluated, alternatively employing water, ethanol/water 50:50, *v/v*, or ethanol/water 30:70, *v/v* (Figure 4b). As expected due to MG solubility, the higher the ethanol percentage in the receiving phase, the faster the MG diffusion. Thus, ethanol/water 50:50, *v/v* was selected, because it is more suitable for assuring the sink condition for MG diffusion [34].

**Figure 5:**
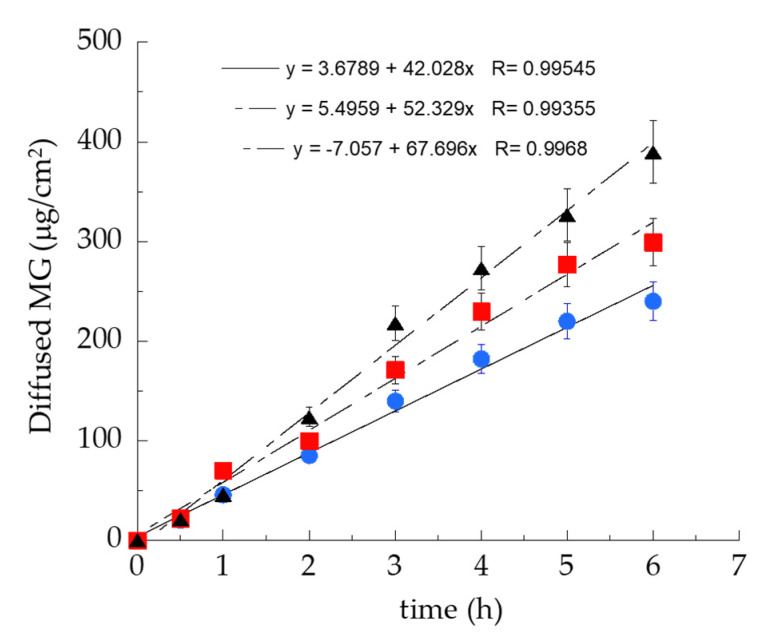
MG diffusion kinetics from Sol-MG (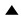), E-MG (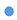), or TE5-MG (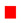), as determined by the Franz cells assembled with the nylon membrane and ethanol/water 50:50 *v/v* as receiving phase. Data are the mean of 6 independent experiments ± s.d.

The MG diffusion kinetics from E-MG, TE5-MG, and Sol-MG were evaluated using Franz cells and the previously selected parameters (Figure 5).

MG diffusion trend was Sol-MG > TE5-MG > E-MG. F values, corresponding to the slopes of the diffusion profiles, and D values, calculated dividing F by MG concentration (mg/mL) in the different forms, are reported in Table 4. Both E-MG and TE5-MG controlled MG diffusion with respect to the simple Sol-MG. Particularly, MG diffusion from TE5-MG was 1.84-fold slower compared to Sol-MG and 1.24-fold faster compared to E-MG. The differences in diffusion found in the case of TE5-MG compared to E-MG could be attributed to the alteration of the packing density of PC bilayers due to TW_80_ positioning, leading to a higher vesicle permeability and resulting in faster diffusion of the entrapped MG. To investigate the biological behavior of E-MG and TE5-MG towards cutaneous inflammation, in vitro experiments were conducted.

### 3.6. Antioxidant and Anti-Inflammatory Effects of MG on HaCaT Cells Exposed to CS

**Figure 6:**
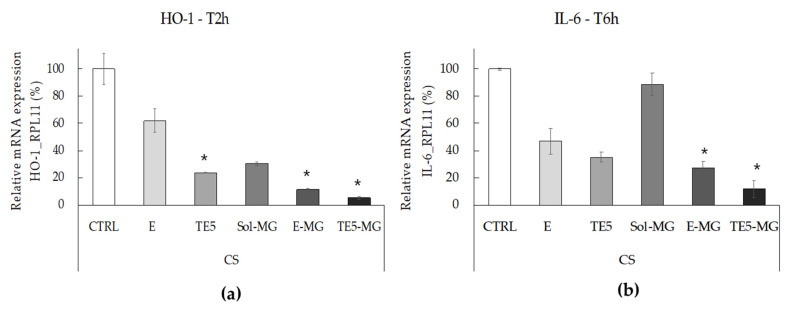
Antioxidant (**a**) and anti-inflammatory (**b**) effect of empty or MG-loaded vehicles on HaCaT cells exposed to CS for 30 min. Transcript levels of HO-1 (**a**) and IL-6 (**b**) were measured using qRT-PCR respectively 2 and 6 h post-exposure. Data are the results of the averages of at least three different experiments ± s.d. * *p* < 0.05 vs. CTRL sample by ANOVA.

The correlation between oxidative stress and inflammation in the skin induced by pollutants has been extensively described in the literature [51,52,53]. Pollutants can initiate oxidative and inflammatory reactions within the cutaneous tissue, where an ox-inflammatory status further induces cutaneous damage [54,55]. To test the protective effect of MG against the cutaneous ox-inflammatory challenges, HaCaT cells were treated with E-MG, TE5-MG, and Sol-MG (MG 10 μM) and then exposed to CS, while untreated cells exposed to CS were taken as the control. Hence, HO-1, a key enzyme involved in cellular oxidative defense, and IL-6, a pro-inflammatory cytokine, were investigated as markers of the oxidative and inflammatory responses, by assessing RT-PCR analyses. As expected, in the case of untreated HaCat cells, the exposure to CS induced a significant increase of HO-1 and IL-6 transcript levels, with respect to untreated cells exposed to filtered air (Supplementary Figure S2). In the case of treated cells, CS was able to induce strong mRNA expression levels of HO-1, 2 h post-exposure, as depicted in Figure 6a. Indeed, pre-treatment with MG formulations significantly prevented CS-induced HO-1 expression in HaCaT cells with respect to untreated cells exposed to CS (CTRL). Notably, this effect was even more pronounced when MG was delivered by both nanosystems with respect to Sol-MG, and particularly evident in the case of TE5-MG. In addition, empty nanosystems, E and TE5, also seemed to exert a protective effect, which can be ascribed to their composition being rich in PC [30]. Indeed, many phospholipids present within the skin have been demonstrated to be target molecules for pollutants, including CS, leading to lipid peroxidation [52,53]. In this context PC externally applied by nanosystems could represent a target for CS, therefore, acting as a protective barrier, or a “sacrificing agent” to the oxidative damage that normally occurs in cells upon pollutants exposure. Notably, the higher protective effect found in the case of TE5 with respect to E could be ascribed to an inhibitory activity on ROS production as previously described for TW_80_ [56]. In this respect, TE5 could exert a certain onward antioxidant effect due to the presence of TW_80_. Furthermore, E-MG and TE5-MG treatments were able to inhibit the inflammatory response triggered by CS in HaCaT cells, 6 h after exposure, as highlighted by the significant lower mRNA expression levels of the pro-inflammatory cytokine IL-6 (Figure 6b) with respect to untreated cells exposed to CS.

Remarkably, Sol-MG did not exhibit any protective effect against the CS-induced inflammatory insult, confirming that E-MG and TE5-MG are more effective in preventing skin damage, especially in the context of the inflammatory response. Particularly, the protective effect was more evident in the case of TE5-MG with respect to E-MG. The differences in antioxidant and anti-inflammatory effects of E and TE5 could be attributed to a more rapid disaggregation of TE5, due to the presence of TW_80_ intercalating within the PC bilayer. Nonetheless, further studies are required to better understand the kinetics of vesicle disaggregation and MG release within cells.

**Figure 7:**
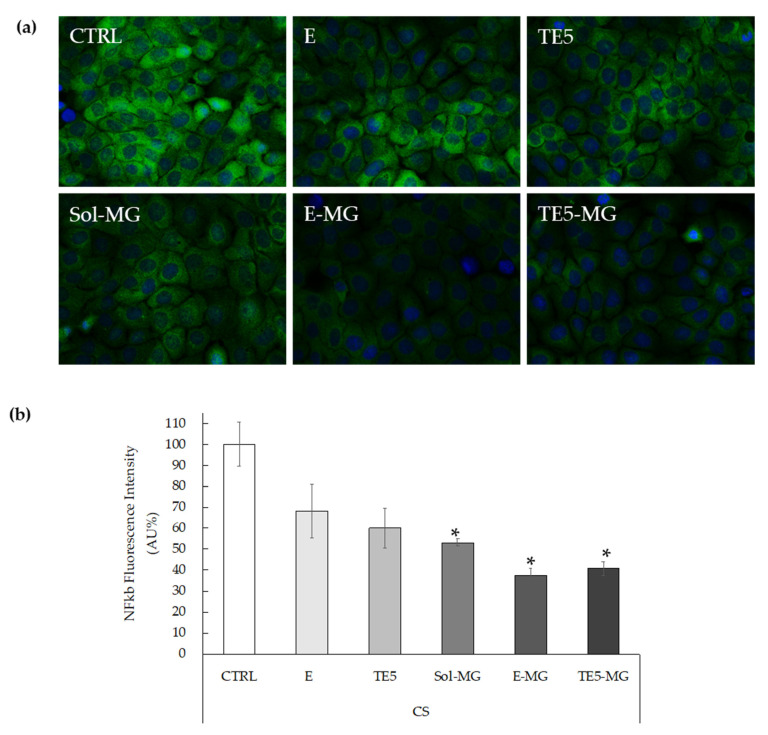
Representative images of immunofluorescence staining for NF-kB (green) and DAPI (blue) at 40× magnification on HaCaT cells treated with empty or MG-loaded vehicles and exposed to CS (**a**). Quantification of immunofluorescence staining of NF-kB 1h after CS exposure (**b**). Data were normalized with respect to the CTRL sample and expressed as arbitrary units ± SD. * *p* < 0.05 vs. CTRL sample by ANOVA.

Moreover, considering that IL-6 is under the transcriptional regulation of NF-kB [57,58,59], immunofluorescence staining was performed to assess the level of NF-kB in HaCaT pre-treated with MG and exposed to CS. In particular, HaCaT cells were treated with E, TE5, E-MG, and TE5-MG, comparing the efficacy of entrapped MG to that of Sol-MG, quantified with respect to untreated cells (CTRL). As shown in Figure 7a, CS exposure clearly induced NF-kB levels in CTRL cells, as evidenced by the green fluorescence staining. In parallel with the IL-6 data, the treatment with E-MG and TE5-MG reduced NF-kB expression by circa 60% in HaCaT (Figure 7b).

Noticeably, the comparison of these data with that of cells treated with Sol-MG, demonstrated that E-MG and TE5-MG exert a stronger anti-inflammatory effect, as depicted by the reduction in green fluorescence intensity. These results corroborate the suitability of E-MG and TE5-MG as antioxidant and anti-inflammatory topical treatments of skin diseases induced by pollutant stressors such as CS.

### 3.7. E and ET Uptake in Keratinocytes Detected by TEM

**Figure 8:**
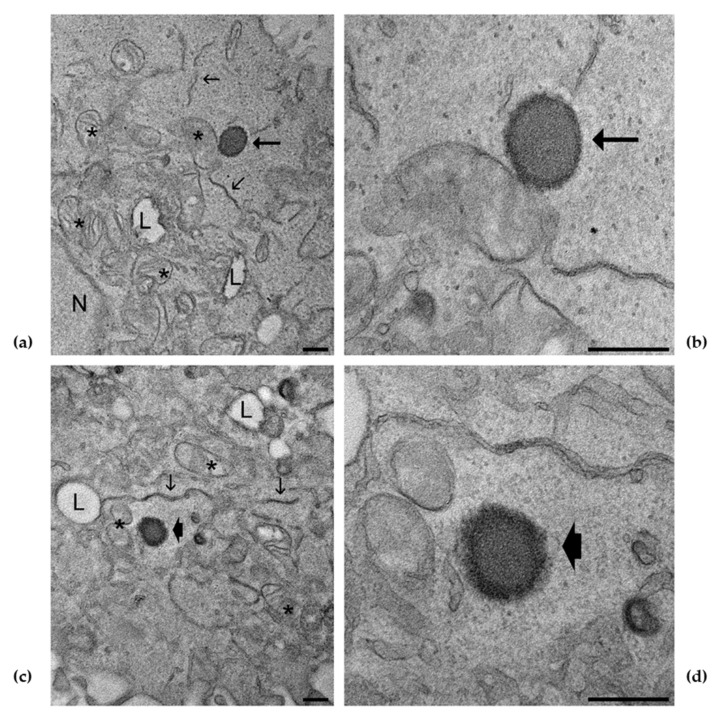
TEM micrographs of keratinocytes treated with E (**a**), high magnification detail in (**b**) and TE5 (**c**), high magnification detail in (**d**). E (arrows) and TE5 (arrowheads) are clearly recognizable in the cytoplasm; note the darker edge likely corresponding to the phospholipid layer. Mitochondria (asterisks), endoplasmic reticulum (thin arrows), lipid droplets (L), nucleus (N). Bars correspond to 200 nm.

TEM analysis, performed to shed light on vesicle interaction with cells, confirmed the uptake of E and TE5 within keratinocytes, and, at different magnifications, the presence of E (Figure 8a,b) and TE5 (Figure 8c,d) is clearly evident. The morphology of the vesicles is comparable to the cryo-TEM images shown in Figure 2, characterized by an external PC layer and an inner core. The ultrastructural features of cell organelles were not altered by the presence of E and TE5, according to MTT data demonstrating good cell viability. These findings corroborate previous studies conducted by many authors, indicating that E and TE vesicles can enter through the cellular membrane, releasing the loaded molecule within cells [11,60,61,62,63]. Moreover, these results clearly demonstrated that both E and TE5 are able to penetrate the cells maintaining their own structure, thus, supporting our starting hypothesis.

## 4. Conclusions

The present investigation demonstrated the suitability of E and TE for MG solubilization and delivery. This preformulatory study has pointed out the influence of E and TE composition on zeta potential, vesicle size distribution, and stability. Strikingly, the results of RT-PCR and immunofluorescence demonstrated that E and TE5 can deliver MG to the target cell, enhancing the keratinocyte antioxidant defense status, while protecting from the cutaneous ox-inflammatory damage induced by CS. These data have been corroborated by TEM analyses, showing the presence of intact vesicles within keratinocytes. Nevertheless, further in vivo studies are required to better elucidate the kinetics of vesicle disaggregation within the cells and the different mechanisms of interaction of E and TE5 with the skin.

## Figures and Tables

**Table 1:** Mangiferin solubility in different solvents and solvent mixtures.

Solvents	MG Solubility (mg/mL)
Water	0.11 ± 0.01
Ethanol	0.72 ± 0.01
Methanol	0.20 ± 0.02
Ethanol/Methanol, 50:50 (*v/v*)	0.21 ± 0.00
Dimethyl sulfoxide	4.00 ± 0.10
Dimethyl sulfoxide/Ethanol, 20:80 (*v/v*)	1.00 ± 0.07
Propylene glycol/Water, 60:40 (*v/v*)	0.30 ± 0.04

**Table 2:** Composition of ethosomes and transethosomes.

Formulation	PC ^1^ % *w/w*	Ethanol % *w/w*	TW_80_^2^ % *w/w*	TW_20_^3^ % *w/w*	SP_80_^4^ % *w/w*	SP_20_^5^ % *w/w*	DDAB ^6^ % *w/w*	MG ^7^ % *w/w*	Water % *w/w*
E	0.9	29.1	-	-	-	-	-	-	70
E-MG	0.9	29.1	-	-	-	-	-	0.1	69.9
TE1	0.89	28.81	-	0.3	-	-	-	-	70
TE2	0.89	28.81	-	-	-	0.3	-	-	70
TE3	0.89	28.81	-	-	0.3	-	-	-	70
TE4	0.89	28.96	0.15	-	-	-	-	-	70
TE5	0.89	28.81	0.3	-	-	-	-	-	70
TE6	0.89	28.51	0.6	-	-	-	-	-	70
TE7	0.9	29.1	-	-	-	-	0.1	-	69.9
TE8	0.9	29.1	-	-	-	-	0.2	-	69.8
TE9	0.9	29.1	-	-	-	-	0.3	-	69.7
TE5-MG	0.9	29.1	0.3	-	-	-	-	0.1	69.6
TE8-MG	0.9	29.1	-	-	-	-	0.2	0.1	69.7

^1^: phosphatidyl choline; ^2^: polysorbate 80; ^3^: polysorbate 20; ^4^: sorbitane monooleate; ^5^: sorbitan monolaurate; ^6^: dimethyldidodecylammonium bromide; ^7^: mangiferin.

**Table 3:** Dimensional distribution parameters of ethosomes and transethosomes, as determined by PCS.

Formulation	Z-Average (nm) ± s.d. (nm)	Typical Intensity Distribution		Dispersity Index ± s.d. (nm)	Z Potential ± s.d. (nm)
		nm	Area %		
E	206.3 ± 33.13	260.7 5065	99.5 0.5	0.146 ± 0.04	−23.39 ± 0.2
TE1	294.25 ± 2.33	318.35 4974	99.3 0.7	0.120 ± 0.01	−10.55 ± 0.4
TE2	411.1 ± 13.72	421.85 4929	99 1	0.177 ± 0.02	−13.45 ± 0.2
TE3	287.2 ± 9.47	288.1	100	0.052 ± 0.03	−18.11 ± 0.4
TE4	288.8 ± 11.31	289.2	100	0.068 ± 0.01	−16.90 ± 0.5
TE5	186.2 ± 20.29	187.0	100	0.131 ± 0.05	−33.56 ± 0.3
TE6	158.1 ± 2.12	159.2	100	0.122 ± 0.02	−14.23 ± 0.5
TE7	127.45 ± 8.84	129.0	100	0.155 ± 0.04	60.53± 0.4
TE8	98.82 ± 0.22	99.2	100	0.103 ± 0.02	67.35 ± 0.5
TE9	82.87 ± 8.89	83.2	100	0.082 ± 0.02	71.92 ± 0.6
E-MG	189.8 ± 13.46	178.8 5049	99.6 0.4	0.134 ± 0.02	−20.58 ± 0.3
TE5-MG	169.3 ± 0.46	168.7	100	0.132 ± 0.02	−28.29 ± 0.4
TE8-MG	86.41 ± 1.09	87.2	100	0.198 ± 0.01	84.08 ± 0.6

s.d.: standard deviation.

**Table 4:** Entrapment capacity, DPPH radical-scavenging capacity, and diffusion coefficients of MG-loaded in the indicated formulations.

Formulation	EC ^a^ (%)	DPPH IC_50_ (µg/mL)	F ^b^ (mg/cm^2^ × h × 10^3^)	D ^c^ (cm/h × 10^3^)
Sol-MG ^d^	-	-	67.70 ± 5.51	96.71 ± 7.87
E-MG	68 ± 3	20.305 ± 1.89	42.03 ± 2.41	42.03 ± 2.41
TE 5-MG	63 ± 2	18.407 ± 1.16	52.33 ± 3.81	52.33 ± 3.81
TE 8-MG	57 ± 1	25.465 ± 0.98	-	-
MG ^e^	-	17.180 ± 0.53	-	-

^a^: Entrapment capacity; ^b^: Diffusion coefficient; ^c^: Flux; ^d^: MG (0.7 mg/mL) in ethanol/water 30:70 (*v/v*); ^e^: MG (1 mg/mL) in dimethyl sulfoxide/ethanol 20:80 (*v/v*); Data are the mean of 6 independent Franz cell experiments.

## Data Availability

Not applicable.

## Supplementary Materials

The following are available online at https://www.mdpi.com/article/10.3390/antiox10050768/s1, Figure S1: Representative images of unloaded (**a**) and MG loaded (**b**) ethosomes and transethosomes; Figure S2: Transcript levels of HO-1 (**a**) and IL-6 (**b**) on HaCat cells exposed to air (Air) or to CS for 30 min. HO-1 and IL-6 were measured using qRT-PCR respectively 2 and 6 h post-exposure. Data are the results of the averages of at least three different experiments ± s.d. * *p* < 0.05 vs. CTRL sample by ANOVA.
