# Supplementary material for: Ethosomes and Transethosomes for Mangiferin Transdermal Delivery
Source: Antioxidants (Basel). 2021 May 12;10(5):768. doi: 10.3390/antiox10050768 (PMC8150765; doi:10.3390/antiox10050768)
Supplement: Supplementary file 1 [file antioxidants-10-00768-s001.zip › antioxidants-1221109-supplementary.pdf]

Supplementary Material

Ethosomes and transethosomes for mangiferin transdermal delivery

Maddalena Sguizzato[1], Francesca Ferrara[2], Supandeep Singh Hallan[1], Anna Baldisserotto[3], Markus Drechsler[4], Manuela Malatesta[5], Manuela Costanzo[5], Rita Cortesi[1], Carmelo Puglia[6], Giuseppe Valacchi[2,7,8*] and Elisabetta Esposito[1*]

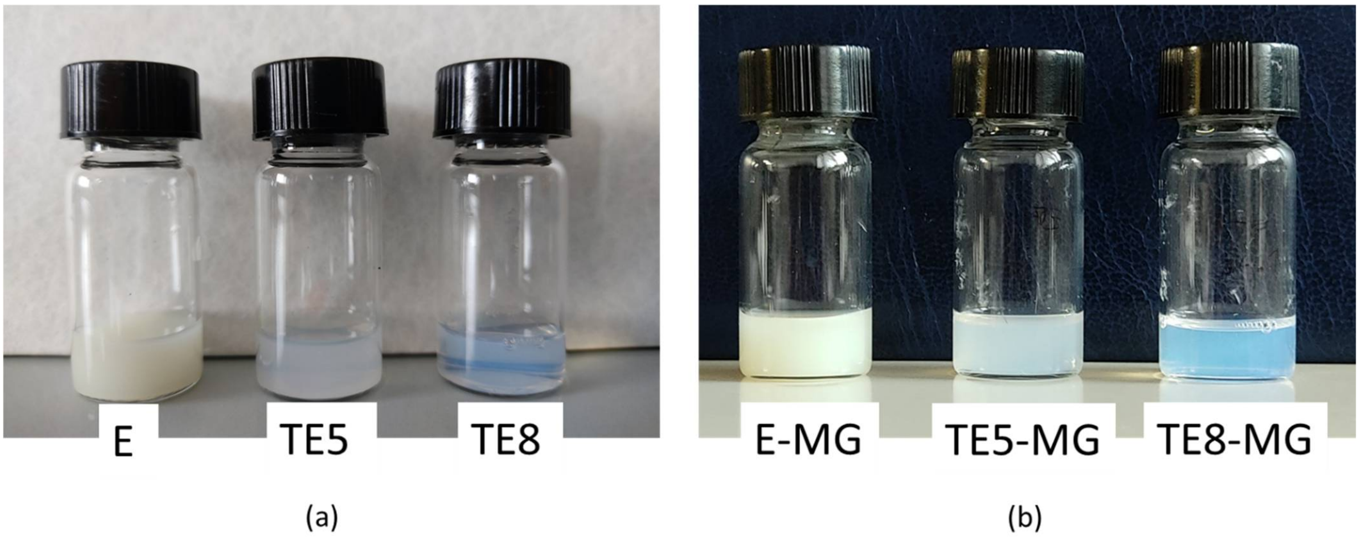

Figure S1. Representative images of unloaded **(a)** and MG loaded **(b)** ethosomes and transethosomes

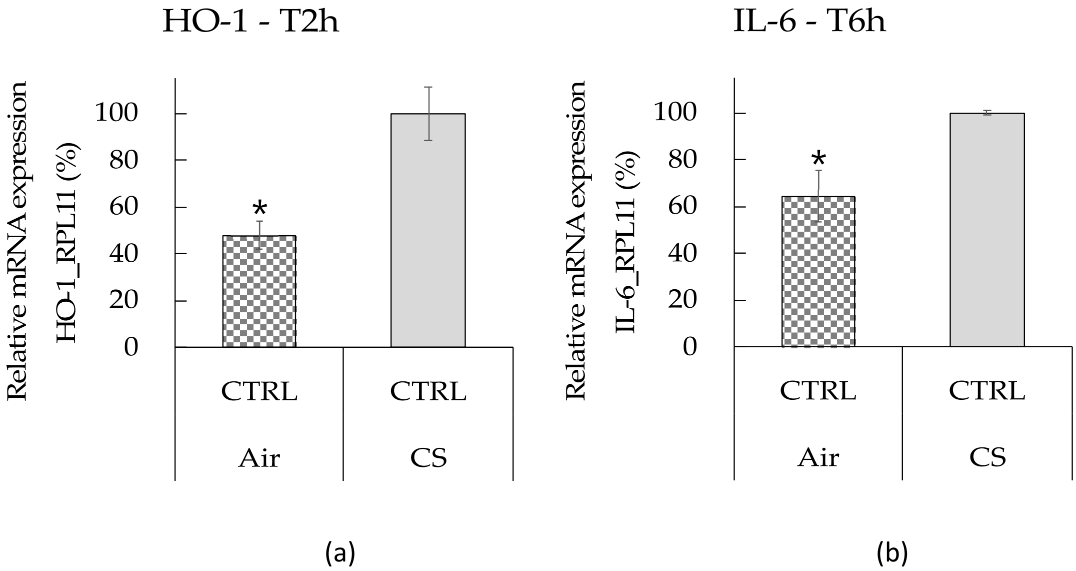

Figure S2. Transcript levels of HO-1 (a) and IL-6 (b) on HaCat cells exposed to air (Air) or to CS for 30 min. HO-1 and IL-6 were measured using qRT-PCR respectively 2 and 6 h post-exposure. Data are the results of the averages of at least three different experiments ± s.d. *$p < 0.05$ vs. CTRL sample by ANOVA.
